# How effective and cost-effective was the national mass media smoking cessation campaign ‘Stoptober’?^☆^

**DOI:** 10.1016/j.drugalcdep.2013.11.003

**Published:** 2014-02-01

**Authors:** Jamie Brown, Daniel Kotz, Susan Michie, John Stapleton, Matthew Walmsley, Robert West

**Affiliations:** a Cancer Research UK Health Behaviour Research Centre, University College London, London WC1E 6BT, UK; b Department of Family Medicine, CAPHRI School for Public Health and Primary Care, Maastricht University Medical Centre, Maastricht, The Netherlands; c Department of Clinical, Educational and Health Psychology, University College London, London, UK; d National Centre for Smoking Cessation and Training, London, UK; e Addictions Department, Institute of Psychiatry, Kings College London, London, UK; f Public Health England, Wellington House, London 9, UK

**Keywords:** Smoking, Cessation, Quitting, Mass media, Cost-effectiveness, Stoptober

## Abstract

**Background:**

A national smoking cessation campaign based on behaviour change theory and operating through both traditional and new media was launched across England during late 2012 (‘Stoptober’). In addition to attempting to start a movement in which smokers would quit at the same time in response to a positive mass quitting trigger, the campaign set smokers the goal of being smoke-free for October and embodied other psychological principles in a range of tools and communications.

**Methods:**

Data on quit attempts were obtained from 31,566 past-year smokers during nationally representative household surveys conducted monthly between 2007 and 2012. The effectiveness of the campaign was assessed by the increase in national quit attempt rate in October relative to other months in 2012 vs. 2007–2011.

**Results:**

Relative to other months in the year, more people tried to quit in October in 2012 compared with 2007–2011 (OR = 1.79, 95%CI = 1.20–2.68). In 2012 there was an approximately 50% increase in quitting during October compared with other months of the same year (9.6% vs. 6.6%; OR = 1.50, 95%CI = 1.05–2.15), whereas in 2007–2011 the rate in October was non-significantly less than in other months of the same period (6.4% vs. 7.5%; OR = 0.84, 95%CI = 0.70–1.00). Stoptober is estimated to have generated an additional 350,000 quit attempts and saved 10,400 discounted life years (DLY) at less than £415 per DLY in the modal age group.

**Conclusions:**

Designing a national public health campaign with a clear behavioural target (making a serious quit attempt) using key psychological principles can yield substantial behaviour change and public health impact.

## 1 Introduction

Smoking is one of the leading risk factors for premature death and disability and is estimated to kill six million people each year (Lim et al., 2012, World Health Organisation, 2012). Mass media smoking cessation campaigns as part of comprehensive tobacco control programmes have been effective in helping to reduce this burden (Bala et al., 2008, Bala et al., 2012). Campaigns can vary in a number of characteristics that may determine their effectiveness (Langley et al., 2013) and studies comparing different message types have found that harm-focused messaging appears more effective in generating quitting cognitions and behaviour than either those focussing on anti-industry or how-to-quit themes (Durkin et al., 2012). However, there appears to have been little research on the effectiveness of campaigns focussing on positive messaging (Durkin et al., 2012). In late 2012 the English Department of Health with input from an academic partner (RW) designed a campaign called ‘Stoptober’ and, rather than focusing on the harms of smoking, it aimed to create a positive mass quitting trigger and actively support a social movement around a very specific activity: stopping smoking for 28 days.

The stimulus for the campaign was twofold: first was the observation that ‘No Smoking Day’ which takes place every year in March throughout England had been shown to generate an estimated 238,000 attempts to stop in a population of 8.5 million smokers, at a cost of around £750,000 (Kotz et al., 2011). No Smoking Day is different from World No Tobacco Day and has a much higher profile in England. It is a national event that aims to help smokers stop by providing a nationally supportive environment and drawing attention to available treatments. Secondly, it was noticed that Autumn (Fall) in England was a fallow period for quitting activity (West and Brown, 2013a). This led to the idea of a national cessation campaign to generate a burst of activity around that time.

The campaign was underpinned by a number of key psychological principles (see Table 1). One of these was the proposal from social contagion theory that one can use messaging to amplify a campaign by normalising a behaviour and turning it into a movement (Einstein and Epstein, 1980, Locher, 2002, Rende et al., 2005, Smith and Christakis, 2008). Use of the theory was suggested by a network analysis of US population data demonstrating that interconnected groups of people tend to stop smoking in concert (Christakis and Fowler, 2008). The name of the campaign was ‘Stoptober’ as a combination of Stop and October and was designed to build engagement by association with other positive, popular national events that have successfully used similar monikers (e.g., ‘Movember’ to promote moustache growing in November and thereby raise awareness of prostate cancer) and to encourage dissemination both by word-of-mouth and social media (Siemens, 2012). The campaign was broadcast through a combination of traditional and new mass media including TV, press, radio and online adverts, public relations messaging, and Facebook and Twitter activity.

**Table 1:** Psychological principles underpinning the different components of Stoptober.

Psychological principle	Relevant components in Stoptober
*Social contagion theory*: Social networks act as conduits for the spread of attitudes and behaviours. Insofar that messaging can convince a crowd of people to focus their attention on the same common event or goal, social networks will then amplify the reach and intensity of the message	The messaging of the campaign called upon all smokers to attempt to stop smoking on the same date. The campaign was named ‘Stoptober’, as a combination of Stop and October, and was designed to build wide engagement with the event from association with other positive, popular national events that have successfully used similar monikers (e.g., ‘Movember’) and to encourage easy dissemination both by word-of-mouth and social media. The campaign was widely broadcast through a combination of traditional and new mass media including TV, press, radio and online adverts, public relations messaging, and Facebook and Twitter activity
*SMART goals*: SMART goals aim to help people achieve a difficult behavioural goal by encouraging them to begin with a very specific intermediary goal, which seems more attainable, and providing the best possible tools to help them attain that goal	Stoptober set people the challenge, or SMART goal, of being smoke-free for 28 days starting on October 1st 2012
*PRIME theory*: Behaviour is determined on a moment-to-moment basis by a wide variety of motivational inputs, from impulses and inhibitory forces, through desires, drives, and emotional states, to evaluations and plans. As a result the motivational system is inherently unstable and requires constant balancing input to maintain a constant pattern of behaviour. Apparently small external triggers can affect a sudden transition in the system. Whether a change is maintained will depend upon balancing the variety of motivational forces determining the behaviour. Thus, interventions to affect change are more likely to be successful if they target the whole motivational system, rather than just some elements of it, and aim to both weaken the motivational forces to engage in a behaviour and create new sources of desire and control to refrain from it	Stoptober provided triggers for smoking cessation by (i) frequent positive messaging encouraging smokers to stop, and (ii) providing an opportunity to do so at the same time as others. The intervention maximised its likelihood of helping smokers to achieve a smoke-free month by providing a wide variety of support including a postal pack for all those who signed up and a wealth of digital tools from an accompanying website offering brief advice on smoking cessation, to peer support via Facebook, a motivational text-messaging programme and an app to provide ongoing encouragement and self-monitoring tools

Another key psychological principle underpinning the campaign was the use of a SMART (Specific, Measurable, Attainable, Realistic and Time-sensitive) goal (Doran, 1981). SMART goals aim to help people achieve a difficult behavioural goal by encouraging them to begin with a realistic intermediary goal, which is objective, well-specified and bound to a particular target date. Stoptober set people the challenge, or SMART goal, of being smoke-free for 28 days starting on October 1st 2012. The call to action was reinforced by the positive messages that smokers achieving this goal would be at least five times more likely than they were at the start to become permanent ex-smokers as a result of having recovered from the worst of the cravings and withdrawal symptoms (West and Stapleton, 2008).

The final key psychological insight arose from PRIME theory. PRIME theory is a comprehensive theory of motivation that argues behaviour is determined on a moment-to-moment basis by a wide variety of motivational inputs, from impulses and inhibitory forces, through desires, drives, and emotional states, to evaluations and plans (West and Brown, 2013b). The theory also proposes that the motivational system is inherently unstable and requires constant balancing input to maintain a constant pattern of behaviour. In the case of cigarette addiction, cessation is most likely to be successful if a range of support is provided that targets the whole motivational system rather than just some elements of it, and aims to both weaken the diverse and powerful motivational forces to engage in smoking and create new sources of desire to refrain from smoking. Therefore, providing a variety of support to help smokers achieve a smoke-free month was fundamental to the campaign. This included a postal support pack for all those who signed up and a wealth of digital tools from an accompanying website offering brief advice on smoking cessation, to peer support via Facebook, a motivational text-messaging programme and an app that aimed to provide ongoing encouragement and self-monitoring tools.

Thus, the two key elements of the campaign were (1) that it would start a national movement in which smokers would stop smoking at the same time by messaging through traditional and new mass media and (2) provide wide ranging support including digital tools to achieve the stated SMART goal to be smoke-free throughout October while broadcasting the positive message that any smoker would be five times more likely to succeed permanently upon realising this goal.

Because of an ongoing national surveillance programme which has assessed the incidence of key smoking cessation activity every month since November 2006 (Fidler et al., 2011), we were in a good position to provide an independent evaluation of the effectiveness of this campaign: we were able to assess the effectiveness of Stoptober by examining the percentage of smokers making a past-month quit attempt in October relative to other months during 2012 as compared with the preceding four years (2007–2011). With some evidence-based assumptions, we could use this evaluation to estimate the public health impact of the campaign in terms of discounted life years (DLY) gained weighted to reflect the different ages of those stopping and calculate a corresponding incremental cost-effectiveness ratio (ICER) using the known costs of Stoptober. Therefore, this study addressed three research questions: (i) How effective was Stoptober in promoting quit attempts?; (ii) How cost-effective was Stoptober in terms of cost per life year gained?; and (iii) What was the public health impact of Stoptober in terms of total life years it is expected to gain?

## 2 Methods

### 2.1 Study design

The effectiveness of Stoptober was assessed by examining the percentage of smokers reporting a past-month quit attempt in a series of monthly cross-sectional household surveys of representative samples of the population of adults in England between 2007 and 2012. The surveys comprise the ongoing ‘Smoking Toolkit Study’ which is designed to provide information about smoking prevalence and behaviour in England. Each month a new sample of approximately 1800 adults aged ≥16 completes a face-to-face computer-assisted survey with a trained interviewer, which is selected using a form of random location sampling. The full methods have been described in detail and shown to result in a sample that is nationally representative in its socio-demographic composition and proportion of smokers (Fidler et al., 2011). Approval was granted by the UCL ethics committee.

### 2.2 Intervention

‘Stoptober’ encouraged smokers to join a mass quit attempt on October 1st 2012 and stay smoke-free throughout October with a variety of support including digital to help them achieve success. The campaign was underpinned by psychological theory as described in the introduction. Support tools to help smokers achieve a smoke-free month included a postal quitting pack and range of digital tools including an accompanying website that offered brief advice on smoking cessation, motivational text-messaging and an app to provide ongoing support and self-monitoring tools. The known costs of Stoptober provided by the Department of Health were £5.8 million. The breakdown of those costs were as follows: Media advertising (televsion, radio, press, digital, outdoor, media partnerships) £3380,000; Public relations activity £70,000; Local and regional activation of the campaign among participating organisations including the national Stop Smoking Services £500,000; Fees for development and fulfilment of all creatives and products including advertising, website, and digital tools £1820,000; Follow on communications £30,000. The Department of Health conceived of Stoptober with input from an academic partner at UCL (RW); those involved at the department now act for an executive agency of the department called Public Health England.

### 2.3 Participants

A total of 31,566 adults aged 16+ who responded to the survey between 2007 and 2012 and reported past-year smoking (daily or occasionally) of either cigarettes including hand-rolled or tobacco of any other kind (e.g., pipe or cigar) without having quit successfully before the last month were included in the study. In England non-daily smoking is far less common (9.1%, 2884/31,566, of the current sample of past-year smokers) than in the US and other EU countries (range of 16%–22%; Bogdanovica et al., 2011, Centers for Disease Control and Prevention, 2009). Table 2 shows the sample characteristics.

**Table 2:** Unweighted socio-demographic characteristics of respondents.

Characteristic	Oct 2012 (*n* = 433)	Jan–Sept & Nov–Dec 2012 (*n* = 4497)	Oct 2007–11 (*n* = 2378)	Jan–Sept & Nov–Dec 2007–11 (*n* = 24,258)
Mean (SD) age†	42.0 (17.8)	42.9 (17.0)	42.8 (16.7)	42.5 (16.6)
% (*N*) Women	54.3 (235)	48.5 (2183)	50.8 (1207)	50.7 (12,287)
% (*N*) Social grade C2DE	73.4 (318)	71.1 (3198)	69.9 (1663)	67.1 (16,272)
Mean (SD) cigarettes per day§	12.3 (8.5)	12.3 (8.6)	13.6 (8.9)	13.4 (8.7)

† Data on age were missing for 200 participants (range of percentage of missing data across the groups was 0.5–0.7%).

§ Data on cigarettes per day were missing for 1020 participants (range of percentage of missing data across the groups was 3.0–3.6%).

### 2.4 Measures

Quit attempts were assessed by a standard question used in the UK: ‘How many serious attempts to stop smoking have you made in the last 12 months? By serious attempt I mean you decided that you would try to make sure you never smoked again. Please include any attempt that you are currently making and please include any successful attempt made within the last year.’ Smokers who reported at least one quit attempt in the past year were asked: ‘How long ago did your most recent serious quit attempt start? By most recent, we mean the last time you tried to quit. (i) In the last week; (ii) More than a week and up to a month; (iii) More than 1 month and up to 2 months; (iv) More than 2 months and up to 3 months; (v) More than 3 months and up to 6 months; (vi) More than 6 months and up to a year and (vii) Don’t know\Can’t remember’. For the purposes of the analysis, participants were classified into two groups depending on whether or not they responded to either item i or ii to indicate they had made a past-month quit attempt.

Additionally, smokers were asked questions that assessed gender, age, social-grade (AB = higher and intermediate professional/managerial, *C*1 = supervisory, clerical, junior managerial/administrative/professional, *C*2 = skilled manual workers, *D* = semi-skilled and unskilled manual workers, *E* = on state benefit, unemployed, lowest grade workers), and the number of cigarettes smoked per day.

### 2.5 Analysis

To assess the overall effectiveness of Stoptober, the interaction between the month of the year (October vs. all other months) and year of the survey (2012 vs. 2007–2011) on the weighted percentage of past-year smokers making a past-month quit attempt (yes vs. no) was examined in a logistic regression model. The nature of a significant interaction was investigated by examining the simple effect of October vs. all other months on past-month quit attempts in logistic regression separately for 2012 and 2007–2011. Data were weighted using the rim (marginal) weighting technique to match English census data on age, sex, and socioeconomic group. An overall figure for the increase in past-month quitting due to Stoptober was estimated by subtracting the difference between the weighted percentage of all smokers reporting a past-month quit attempt in October 2012 and all other months in 2012 from the equivalent figure for 2007–2011.

To explore whether Stoptober brought forward other quit attempts that would have occurred in the coming months, the effect of the year of the survey (2012 vs. 2007–2011) on the weighted percentage of past-month quit attempts during the months of November and December was examined in a separate logistic regression model. The validity of weighting the data was explored in a sensitivity analysis that repeated the analysis on unweighted data adjusted for differences in smoking and socio-demographic characteristics. To explore the effectiveness of Stoptober in different sociodemographic groups, the 3-way interaction between the month of the year (October vs. all other months), year of the survey (2012 vs. 2007–2011) and sex, social grade or age group on the unweighted percentage of past-month quit attempts were each examined separately in logistic regression models.

For the cost-effectiveness analysis, incremental cost-effectiveness ratios (ICER) of Stoptober were estimated for the total population and separately for four distinct age groups <35 years, 35–44 years, 45–54 years and 55–64 years. An ICER was estimated by using a published standard model for calculating the cost-effectiveness of smoking interventions (Stapleton and West, 2012). The ICER was derived as the cost of Stoptober per smoker divided by the attributable DLY gained per smoker. Quality adjusted LY are typically preferred to LY in cost effectiveness analyses of health interventions. Although in smoking cessation quality adjusted LY typically exceed LY gained because cessation substantially reduces morbidity in addition to mortality, the precise weighting is controversial and varies considerably between researchers (0.9–1.4, Stapleton and West, 2012). Therefore, the model recommends reporting the ICER using the conservative unit of LY gained (i.e., effectively a weight of 1, which allows other authors to adjust the estimates easily by their preferred weight).

The model also recognises that the age at which smokers stop determines the LY gained with the age groups <35 years, 35–44 years, 45–54 years and 55–64 years estimated to gain 10, 9, 6 & 3 undiscounted years of life, respectively (Doll et al., 2004). It is standard practice to discount these LY to reflect the reduced value of extended LY being realised in the future rather than immediately (cf. Lazaro, 2002, van Hout, 1998). Thus, the model discounts the benefit by 3.5% each year between the time of the intervention until the expected age of death of a non-smoker to produce DLY gained of 1.67, 2.15, 2.00 and 1.41 for the four age groups, respectively (Stapleton and West, 2012). Despite the recent NICE recommendation to use a 1.5% rate for interventions that confer health benefits sustained over a long period (National Institute for Health and Clinical Excellence, 2011), the 3.5% rate of discount benefits from being conservative and widely used (Stapleton and West, 2012). To adjust for the ‘natural’ cessation rate expected over the life of a smoker, the model assumes a standard 2.5% annual cessation rate. Accumulated over a lifetime, an annual rate of 2.5% means that a substantial proportion of the smokers quitting during the intervention would have stopped anyway: 66%, 57%, 46%, and 33%, respectively. However, the associated delay in stopping – on average the new age of cessation would be halfway between the intervention age and expected age of death – means that only a proportion of the LY gained are retained from ‘stopping anyway’: 53.0%, 46.4%, 46.1%, and 52.6%, respectively. The product of these figures is the proportion by which the attributable LY should reduce (35.1%, 26.3%, 21.0%, and 17.5%) and results in final figures for the age groups <35 years, 35–44 years, 45–54 years and 55–64 years of 1.08 1.59 1.58 and 1.16 DLY gained attributable to Stoptober per smoker stopping permanently in response to the intervention (Stapleton and West, 2012). In order to estimate an equivalent figure for the total population examined in the current study, an overall figure of 1.18 was derived as the mean of the other figures weighted for smoking prevalence in the different age groups (24%, 25%, 21%, 17% and 10% based on 2012 data from the Smoking Toolkit Study) and the relative proportion of these age groups in the English population (0.31, 0.18, 0.17, 0.14 and 0.20, Office for National Statistics, 2011).

The modelling for the current paper deviates from the published model by adapting the adjustment for relapse (Stapleton and West, 2012). The published model only recommends a method of adjusting sustained cessation rates at 6- and 12-month follow-ups for future relapse and this cannot be directly applied to the current index of effectiveness, i.e., past-month quit attempts. Instead, as with other recent cost-effectiveness analyses that relied on the assessment of past-month quitting, we conservatively estimated that 2.5% of these quit attempts would result in permanent success (Kotz et al., 2011). The other estimates used for the cost-effectiveness modelling are (i) the 8.5 million smokers calculated to be in England at the time of Stoptober (based on 42,467,400 adults aged 16+ (Office for National Statistics, 2011)) and a smoking prevalence of 20% (based on 2012 data from the Smoking Toolkit Study) and (ii) the £5.8 million known costs of Stoptober provided by the Department of Health. All estimates used for the analysis are provided in Table 3.

**Table 3:** Calculations to estimate the cost effectiveness (ICER) and public health impact (attributable DLY gained) of Stoptober across age bands.

	<35	35–44	45–54	55–64	>65	Total
Estimates						
A. % Stoptober effect size (95%CI)	4.15 (0.94–7.37)	4.15 (0.94–7.37)	4.15 (0.94–7.37)	4.15 (0.94–7.37)	4.15 (0.94–7.37)	4.15 (0.94–7.37)
B. Proportion quitting permanently	0.025	0.025	0.025	0.025	0.025	0.025
C. DLY gained attributable to Stoptober per smoker stopping permanently†	1.084	1.588	1.577	1.161	0.000	1.180
D. £million Known costs of Stoptober	5.8	5.8	5.8	5.8	5.8	5.8
E. Total smokers in England	8493,480	8493,480	8493,480	8493,480	8493,480	8493,480
F. Relative proportion of smokers in age band in England§	0.375	0.217	0.180	0.125	0.103	1.000
ICER calculation						
G. (*A* × *B*)% Stopping permanently as a consequence of Stoptober (95%CI)	0.104 (0.023–0.184)	0.104 (0.023–0.184)	0.104 0.023–0.184)	0.104 (0.023–0.184)	0.104 0.023–0.184)	0.104 (0.023–0.184)
H. ([*C* × *G*]/100) × 10^−3^ Mean attributable DLY gained per smoker (95%CI)	1.13 (0.25–2.00)	1.65 (0.37–2.92)	1.64 (0.37–2.90)	1.21 (0.27–2.14)	0.00 (0.00–0.00)	1.22 (0.28–2.17)
I. (*D*/*E*) £ Cost per smoker	0.68	0.68	0.68	0.68	0.68	0.68
J. (I/H) £ ICER (95%CI)	606.87 (137.10–1076.65)	414.26 (93.59–734.94)	417.15 (94.24–740.07)	566.63 (128.01–1005.25)	–	557.70 (125.99–989.41)
Public health impact calculation						
K. (*E* × *F*) Smokers in age band in England	3186,355.35	1842,526.43	1526,013.22	1061,366.30	877,218.70	8493,480.00
L. (*G* × *K*) Smokers stopping permanently in England as a result of Stoptober in age band (95%CI)	3307.56 (747.21–5867.92)	1912.62 (432.08–3393.16)	1584.06 (357.85–2810.27)	1101.74 (248.89–1954.59)	910.59 (205.71–1615.47)	8816.57 (1991.74–15,641.40)
M. (*C* × *L*) Attributable *DLY* gained (95%CI)	3585.40 (809.97–6360.83)	3037.23 (686.14–5388.33)	2498.07 (564.34–4431.80)	1279.12 (288.96–2269.28)	0.00 (0.00–0.00)	10,399.82 (2349.41–18,450.23)

*NB:* In the table figures are rounded but precise figures were used for all calculations. A modifiable excel version of this table is available in the online supplementary materials.

† These figures are taken from an existing model for calculating the cost-effectiveness of smoking interventions (Stapleton and West, 2012). The ‘Total’ figure is the mean of the other figures weighted for smoking prevalence in the different age groups and relative proportion of these age groups in the English population.

§ Estimated from the smoking prevalence figures of 24%, 25%, 21%, 17% and 10% for the different age bands (on the basis of prevalence data from the Smoking Toolkit Study, (Fidler et al., 2011)) and the relative proportion of these age groups in the English population of 0.31, 0.18, 0.17, 0.14 and 0.20 (Office for National Statistics, 2011).

The modelling of these estimated figures to produce the ICERs are shown in the first column of Table 3. In brief, the proportion stopping permanently as a consequence of Stoptober was the product of the Stoptober effect size and the estimate of the percentage who quit permanently. This figure was combined with the established estimates of DLY gained attributable to an intervention per smoker stopping permanently to produce the mean attributable DLY gained per smoker, which was divided by the cost per smoker (known cost/total smokers) to provide the final ICERs. Sensitivity analyses were conducted to examine the effect of modelling different adjustments for relapse, either 1.5% or 3.5%, on the estimated ICERs.

The public health impact of the campaign was estimated in terms of the numbers of DLY gained by all smokers across England (see first column of Table 3). First, the smokers in each age band in England were estimated from the smoking prevalence figures for the different age bands and the relative proportion of these age groups in the English population. The product of these figures and the proportion stopping permanently as a result of Stoptober were calculated before being multiplied by the DLY gained attributable to Stoptober per smoker stopping permanently for each age group to provide the final estimates of the number of DLY gained by smokers in England. This modelling conservatively assumed that there are no LY gained in those quitting who are aged 65 years old and over. This is conservative but necessary in the absence of high quality epidemiological estimates for the magnitude of the years gained for this group.

Alpha was set at *p* < 0.05. To detect a simple effect of October vs. all other months in 2012 on past-month quit attempts of similar size to No Smoking Day (9% vs. 6%) (Kotz et al., 2011), the obtained sample provided 71% power.

## 3 Results

### 3.1 Effectiveness

**Fig. 1:**
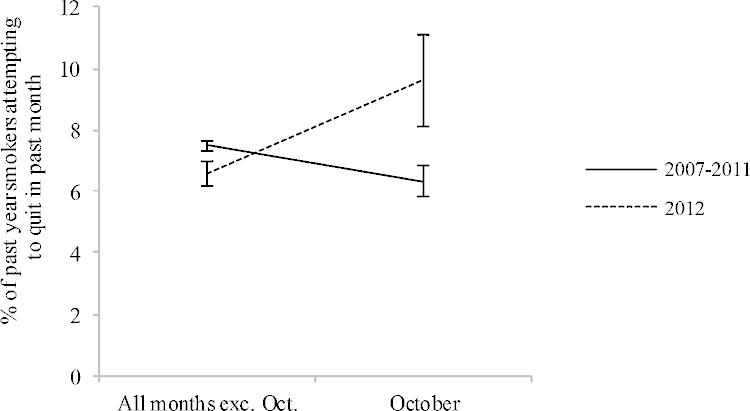
Weighted percentage of past-year smokers who attempted to quit in the past-month during October compared with all other months of the same year for the year 2012 and the aggregated years 2007–2011. The national smoking cessation campaign ‘Stoptober’ was conducted during 2012. Error bars are standard error of the mean.

The weighted percentage of past-year smokers reporting a past-month quit attempt during different months and years of the survey is shown in Fig. 1. These data suggest that during 2012, past-month quit attempts were higher in October than in all other months whereas during the aggregated years 2007–2011 past-month quit attempts were lower in October than all other months. In a logistic regression model, there was an interaction between the month (October vs. all months) and year of survey (2012 vs. 2007–2011) on the percentage of smokers attempting to quit in the past-month (OR = 1.79, 95%CI = 1.20–2.68).

Simple effects revealed that in 2012 there was an increase in past-month quitting during October as compared with all other months (9.6% vs. 6.6%; OR = 1.50, 95%CI = 1.05–2.15), whereas in 2007–2011 there was a non-significant decrease during October (6.4% vs. 7.5%; OR = 0.84, 95%CI = 0.70–1.00). Thus, by subtracting the difference between the weighted percentage of all smokers reporting a past-month quit attempt in October 2012 and all other months in 2012 from the equivalent figure for 2007–2011, the overall estimate of additional past-month quitting attributed to Stoptober was calculated to be 4.15% (95%CI = 0.94–7.37).

In a logistic regression model of the effect of the year of the survey (2012 vs. 2007–2011) on the weighted percentage of past-month quit attempts during the months of November and December, there was no evidence that Stoptober ‘brought forward’ quitting by reducing quit attempts in November and December 2012 as compared with November and December 2007–2011 (6.5% vs. 6.3%; OR = 1.03, 95%CI = 0.75–1.40).

In a sensitivity analysis that used unweighted data and instead adjusted for the characteristics presented in Table 2, the interaction between month (October vs. all months) and year of survey (2012 vs. 2007–2011) remained in a logistic regression model (OR = 1.52, 95%CI = 1.02–2.27). There was no evidence that the effectiveness of Stoptober varied across the social spectrum: there were no 3-way interactions between the month of the year (October vs. all other months), year of the survey (2012 vs. 2007–2011) and social grade (ABC1 vs. C2DE; OR = 1.70, 95%CI = 0.74–3.94), sex (men vs. women; OR = 1.31, 95%CI = 0.60–2.83) or age (<41 years old vs. ≥41 years old; OR = 1.09, 95%CI = 0.49–2.43) on the unweighted percentage of past-month quit attempts in separate logistic regression models.

### 3.2 Cost effectiveness

From the effectiveness analysis, the overall estimate of additional past-month quitting attributed to Stoptober was calculated to be 4.15% (95%CI = 0.94–7.37). The cost-effectiveness analysis using this estimate and the modelling described in the methods is presented in Table 3. The intervention was most cost-effective for the modal 35–44-year-old group with an ICER of £414.26 (95%CI = 93.59–734.94), while the intervention was least cost-effective for the <35 years with an ICER of £606.87 (95%CI = 137.10–1076.65). The ICER for the total population was £557.70 (95%CI = 125.99–989.41).

Sensitivity analyses to examine the influence of the assumption about long-term success rates yielded ICERs for <35 years, 35–44-year-, 45–54 years, 55–64 years and overall, respectively, for 1.5% of £1011.46 (95%CI = 228.50–1794.42), £690.44 (95%CI = 155.98–1224.91), £695.26 (95%CI = 157.06–1233.45), £944.38 (95%CI = 213.34–1675.41) and £929.50 (95%CI = 209.98–1649.02); and for 3.5% of £433.48 (95%CI = 97.93–769.04), £295.90 (95%CI = 66.85–524.96), £297.97 (95%CI = 67.31–528.62), £404.73 (95%CI = 91.43–718.03), and £398.36 (95%CI = 89.99–706.72).

### 3.3 Public health impact

The public health impact of Stoptober is presented in Table 3. On the basis of an effect of Stoptober of 4.15% (95%CI = 0.94–7.37) and the modelling described in the methods, the number of smokers stopping permanently as a result of Stoptober is 8816.57 (95%CI = 1991.74–15,641.40), which is derived from an estimated 352,662.86 quit attempts (95%CI = 79,669.67–625,656.06) succeeding at 2.5% and corresponds to an attributable DLY gained of 10,399.82 (95%CI = 2349.41–18,450.23). This modelling involved the assumption that the effect occurred equally across the age groups, which was reasonable in the absence of evidence to the contrary.

## 4 Discussion

Stoptober appears to have led more than a third of a million smokers to try to quit in October 2012 than would otherwise have done. With multi-faceted public health campaigns it is never possible to isolate any one active ingredient or be absolutely sure about a causal association, but in this case running a campaign based on psychological theory that deviated from the usual approach by providing a positive mass quitting trigger and setting a specific goal of being smoke-free for a month was a calculated risk that appears to have paid off.

As a public health campaign the cost effectiveness of Stoptober compared favourably with other estimates concerning UK anti-tobacco campaigns, which have ranged between £40 and £2000 per discounted life year gained (Kotz et al., 2011, Raikou and McGuire, 2008). With regards to pharmacological interventions for smoking cessation, the cost-effectiveness estimates for Stoptober are approximately 20% of similar measures for NRT or bupropion when they are offered in addition to brief advice (NRT: $3455; bupropion:$2150; both:$2836) or 50% when offered in addition to more intensive counselling (NRT: $1441; bupropion:$920; both $1282; Song et al., 2002). However, it is important to note that any apparent differences in the ICERs between different interventions may actually relate to differences in the methodology for deriving the ICERs. For example, the assessment by Kotz and colleagues examined the impact of a particular campaign (‘No Smoking Day’) using a similar methodology to the current study involving the direct measurement of cost and quitting behaviour; however, the study by Raikou and McGuire estimated expected cost and reductions in adult prevalence from the wider literature as key inputs for a cohort simulation model, while Song and colleagues conducted a literature review of studies reporting the cost-effectiveness of certain smoking cessation medications and used a decision analytic model to produce relative estimates of the cost-effectiveness of different treatments.

In England, the predominant themes of mass media cessation campaigns have been negative harm-focussed and how-to-quit (Langley et al., 2013). While there remains evidence for the effectiveness of negative messaging for promoting smoking cessation (Durkin et al., 2012), the current findings imply that the use of positive messaging could form a more central part of an effective tobacco control mass media strategy. In the wider context, the success of Stoptober should act as an impetus for agencies designing public health campaigns to think more broadly about behaviour change theory and go beyond the prevailing approaches. Michie et al. (2011) have identified nine types of intervention function that can be used on their own or in combination in the design of behaviour change interventions: education, persuasion, incentivisation, coercion, training, restriction, environmental restructuring, modelling and enablement. They also provide a system (COM-B) for analysing what needs to be changed in terms of capability, opportunity and/or motivation to achieve desired behaviour change and using this to select potentially useful intervention functions. It seems that national communication strategies can go further in exploiting these behaviour change pathways than is often assumed to be the case.

Health inequality is a priority and it is important to assess the impact that new interventions have on different social groups (Di Cesare et al., 2013, Marmot, 2013). Stoptober appears to have been equally effective across the social spectrum: there was no evidence of an interaction between the effect of Stoptober and sex, age or social grade. Given the digital component of the campaign, this finding is somewhat surprising. Among smokers, as with the wider population, access to the internet is divided according to affluence and education (Dutton et al., 2007, Stoddard and Augustson, 2006). It may have been that these access inequalities were masked in the current study by smokers across the social grade being equally interested in digital cessation support (Brown et al., 2013). However, it should be noted that the power to detect these 3-way interactions was low, and consequently future research should continue to investigate possible inequalities in the impact of Stoptober, and insofar that they exist, seek refinements to the campaign to mitigate any inequality.

An important strength of this study is that the current analyses were based on data from independent tracking surveys of representative samples of the population in England that asked questions that made no explicit reference to the Stoptober campaign. Additionally, quitting was assessed for several months after the campaign had finished and therefore the analyses included any deflationary impact there may have been on quitting as a consequence of the campaign harvesting attempts that would have occurred regardless. However there are also limitations that must be considered. For example, the cost-effectiveness analysis included only direct costs as the additional indirect and opportunity costs for the Department of Health would have been extremely difficult to estimate accurately. Against this limitation of under-estimating associated cost, the majority of the other assumptions were conservative in the interests of comparability, such as the exclusion of any estimate of either the reduction in morbidity that results from smoking cessation or the DLY gained by those who quit aged 65 years and above, and the use of a 3.5% discount rate as recommended by the published model on which the estimates were based (Stapleton and West, 2012). The reason that 3.5% is conservative is that there has been a recent NICE recommendation to use a 1.5% rate for interventions that confer health benefits sustained over a long period (National Institute for Health and Clinical Excellence, 2011), and the use of a smaller discounting rate would have led to higher estimates of the DLY saved and correspondingly lower ICERs. A second limitation is that there is a possibility that some respondents misclassified their past month quitting. However, the risk of misclassification is no more likely for any particular month because the question was precisely the same for all months included in the analysis and asked without any reference to Stoptober. As a result, the possibility of misclassification does not undermine the finding that, relative to other months in the year, more people tried to quit in October 2012 as compared with 2007–2011 but it may have created noise leading to imprecision in the estimate of the effect. A third limitation is that the increased quitting was not directly attributed to varying degrees of exposure to the campaign. Typically, mass media campaigns are evaluated by directly assessing and relating exposure to the campaign to an outcome indicative of campaign activity (Durkin et al., 2012). Instead, this study constituted a natural experiment in which it was assumed there was only high and low exposure and used long-term tracking data to provide a reliable baseline for low exposure under temporally similar conditions. Finally, our survey only measured additional quitting in England, and although the campaign only targeted England directly, there was almost certainly a positive related effect of the campaign on quitting in other countries of the United Kingdom that was not included in the modelling.

On the basis of the current evaluation, and internal ones conducted by the Department of Health, the Stoptober campaign is planned to run again in 2013, and is likely to become a permanent campaign contingent upon its continued effectiveness. In order to establish whether Stoptober remains a success–alternatively the campaign may have benefitted from novelty or be found to suffer from ‘burnout’—an ongoing assessment of the effectiveness of Stoptober is important.

In conclusion, the national ‘Stoptober’ mass media smoking cessation campaign featuring digital support appears to have provided excellent value for money as a life-saving public health intervention. Designing a national public health campaign with a clear behavioural target (making a serious quit attempt) using key psychological principles can yield a substantial return in terms of behaviour change and public health impact.

## Role of funding source

Funding was provided for the conduct of this research and preparation of the manuscript. The funders had no final role in the study design; in the collection, analysis and interpretation of data; in the writing of the report; or in the decision to submit the paper for publication. All decisions taken by the investigators were unrestricted.

## Contributors

JB & RW conceived of the design of the current study. JB performed the data analysis and interpretation with input from RW. JB drafted the paper and all other authors provided critical revisions. All authors approved the final version of the paper for submission.

## Conflicts of interest

JB & DK have received unrestricted research grants from Pfizer. RW undertakes research and consultancy and receives fees for speaking from companies that develop and manufacture smoking cessation medications (Pfizer, J&J, McNeil, GSK, Nabi, Novartis, and Sanofi-Aventis). He also has a share of a patent for a novel nicotine delivery device. MW worked for the Department of Health, and now works for their executive agency Public Health England; the Department of Health with input from RW conceived, and then developed and implemented Stoptober. SM & JAS have no conflicts.
